# The genome sequence of the liver fluke *Opisthorchis viverrini *(Poirier, 1886) Stiles & Hassall, 1896

**DOI:** 10.12688/wellcomeopenres.23535.1

**Published:** 2025-01-10

**Authors:** Arporn Wangwiwatsin, Siriyakorn Kulwong, Chitsakul Phuyao, Attapol Titapun, Watcharin Loilome, Poramate Klanrit, Nisana Namwat, Paiboon Sithithaworn, Stephen R. Doyle, Matthew Berriman, Thomas Crellen, Wellcome Sanger Institute Tree of Life Management, Samples and Laboratory team, Wellcome Sanger Institute Scientific Operations: Sequencing Operations, Wellcome Sanger Institute Tree of Life Core Informatics team, Tree of Life Core Informatics collective

**Affiliations:** 1 Department of Systems Biosciences and Computational Medicine, Faculty of Medicine, Khon Kaen University, Khon Kaen, Thailand; 2 Cholangiocarcinoma Research Institute, Khon Kaen University, Khon Kaen, Thailand; 3 Department of Surgery, Faculty of Medicine, Khon Kaen University, Khon Kaen, Thailand; 4 Department of Parasitology, Khon Kaen University, Khon Kaen, Thailand; 5 Wellcome Sanger Institute, Hinxton, England, UK; 6 School of Infection & Immunity, University of Glasgow, Glasgow, Scotland, UK; 7 School of Biodiversity One Health and Veterinary Medicine, University of Glasgow, Glasgow, Scotland, UK; 8 Big Data Institute, Li Ka Shing Centre for Health Information and Discovery, University of Oxford, Oxford, England, UK

**Keywords:** Opisthorchis viverrini, liver fluke, genome sequence, chromosomal, Opisthorchiida, neglected tropical diseases, foodborne trematodiasis

## Abstract

We present a genome assembly from a specimen of *Opisthorchis viverrini* (liver fluke; Platyhelminthes; Trematoda; Opisthorchiida; Opisthorchiidae). The genome sequence has a total length of 627.20 megabases. Most of the assembly (97.89%) is scaffolded into 6 chromosomal pseudomolecules. The mitochondrial genome has also been assembled and is 18.04 kilobases in length.

## Species taxonomy

Eukaryota; Opisthokonta; Metazoa; Eumetazoa; Bilateria; Protostomia; Spiralia; Lophotrochozoa; Platyhelminthes; Trematoda; Digenea; Opisthorchiida; Opisthorchiata; Opisthorchiidae; *Opisthorchis*; *Opisthorchis viverrini* (Poirier, 1886) Stiles & Hassall, 1896 (NCBI:txid6198)

## Background

*Opisthorchis viverrini*, commonly known as the Southeast Asian liver fluke, is a species of trematode with a complex parasitic lifecycle which includes molluscan and piscine intermediate hosts and a mammalian definitive host. Since 1994 the parasite has been classified as carcinogenic to humans by the International Agency for Research on Cancer ( IARC Working Group on the Evaluation of Carcinogenic Risks to Humans, 1994). This parasite is a direct cause of bile duct cancer (cholangiocarcinoma; CCA), which is supported by both epidemiological evidence, as there is geographical overlap between the distribution of *O. viverrini* infection and an elevated incidence of CCA, and empirical laboratory studies where infection with the parasite induces CCA in hamster models ( Elkins *et al.*, 1990; Haswell-Elkins *et al.*, 1994; Loilome *et al.*, 2006; Ohshima *et al.*, 1994; Pinlaor *et al.*, 2004a, Pinlaor *et al.*, 2004b; Yongvanit *et al.*, 2012). The parasite is prevalent throughout Southeast Asia, where consumption of dishes containing raw freshwater fish is a cultural tradition, and it is estimated to infect 12 million people across Thailand, Cambodia, Lao PDR and Vietnam ( Zhao *et al.*, 2021). However, *O. viverini* also poses risks to travellers who may contract the parasite, either through direct consumption or via contamination of surfaces or cooking utensils ( Grundy-Warr *et al.*, 2012; Shin *et al.*, 2010). The morbidity resulting from infection with *O. viverrini* is considered a Neglected Tropical Disease under the category of ‘Foodborne Trematodiasis’ ( Borlase *et al.*, 2023). The incidence of bile duct cancer in humans is notably higher in *O. viverrini*-endemic areas ( Florio *et al.*, 2020), and is a major burden in terms of the cost to both healthcare systems and socioeconomically, as it typically afflicts the highest earning family members ( Khuntikeo *et al.*, 2018).

Humans contract *O. viverrini* by consuming raw freshwater fish of the family Cyprinidae (carp), which are encysted with the infective stage of the parasite (metacercariae). Adult *O. viverrini* reside in the bile ducts of mammalian definitive hosts, where they can survive for over a decade ( Crellen *et al.*, 2024). As a hermaphroditic species, each adult worm possesses both male and female reproductive organs. Upon reaching reproductive maturity, eggs are released into the intestine and subsequently expelled into the environment with faeces. In areas with poor sanitation, these eggs contaminate natural water sources, where they are ingested by *Bithynia* snails. Within the snail host, the eggs hatch and develop into larval stages (miracidia, redia, and cercariae) and multiply asexually. Cercariae exit the snails as free-swimming cercariae before seeking out a piscine host and penetrating the skin, then encysting into metacercariae within fish muscle. Metacercariae are then inadvertently consumed by mammalian definitive hosts including humans and, potentially, domestic cats and dogs ( Harinasuta & Harinasuta, 1984; Sota *et al.*, 2022). In cases where the tumour is resectable, surgeons in Thailand have encountered multiple instances where adult worms were present inside the bile ducts of cancer patients, and they were removed along with the tumour and adjacent margin areas. Such cases have become less common in Thailand due to parasite control programs ( Sithithaworn *et al.*, 2014). However, they may still occur in other endemic areas lacking adequate public health measures.

Here, we present a chromosome-level reference genome for *O. viverrini* containing six autosomal chromosomes and a complete mitochondrial genome. We used long-read sequencing and Hi-C chromatin conformation on two adult worms obtained from human patients in Thailand. The *O. viverrini* worms were recovered from the bile ducts of cancer patients undergoing curative surgery during the course of treatment, by a team of Hepato-Pancreato-Biliary surgeons led by Prof. Narong Khuntikeo at Srinakarind Hospital in Thailand. The specimens used here were held by, and accessed from, the biobank of the Cholangiocarcinoma Research Institute at Khon Kaen University.

The availability of this new genome data represents a significant improvement over a previous draft genome ( Young *et al.*, 2014), which relied solely on short-read sequencing and used pooled DNA from multiple adult worms which were collected as metacercariae from fish, rather than human patients. The circularised mitochondrial genome also replaces a previous incomplete version ( Cai *et al.*, 2012). This, greatly improved, reference for *O. viverrini* will provide an essential foundation for functional genomics aimed at developing diagnostics and therapeutics against the parasite, as well as enabling genomic epidemiology to inform evidence-based disease control interventions across Southeast Asia ( Crellen *et al.*, 2021).

## Genome sequence report

**Figure 1.**
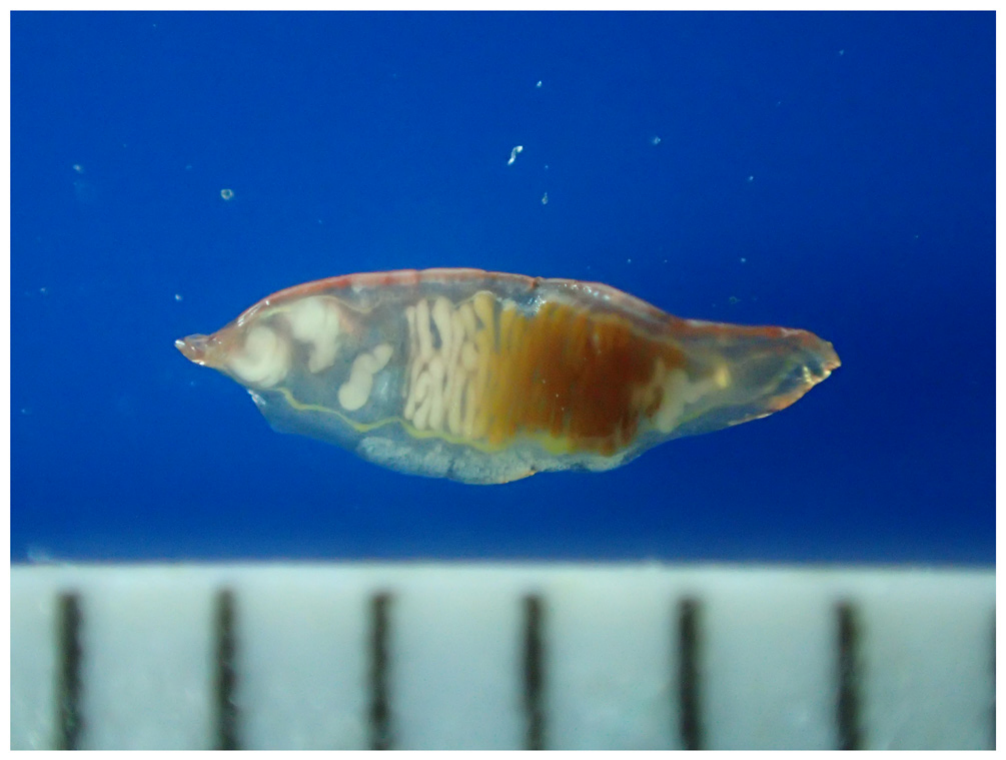
Adult *O. viverrini* acquired from a resected bile duct cancer case. (Not the specimen used for this genome sequencing). Photo taken by Mr. Chitsakul Phuyao, Cholangiocarcinoma Research Institute, Khon Kaen University.

The genome of *Opisthorchis viverrini* ( Figure 1) was sequenced using Pacific Biosciences single-molecule HiFi long reads, generating a total of 22.18 Gb (gigabases) from 2.90 million reads, providing an estimated 32-fold coverage. Primary assembly contigs were scaffolded with chromosome conformation Hi-C data, which produced 127.26 Gb from 842.81 million reads. Specimen and sequencing details are summarised in Table 1.

**Table 1.** Specimen and sequencing data for *Opisthorchis viverrini*.

Project information			
**Study title**	Opisthorchis viverrini (Asian liver fluke)		
**Umbrella BioProject**	PRJEB67413		
**Species**	*Opisthorchis viverrini*		
**BioSample**	SAMEA110137926		
**NCBI taxonomy ID**	6198		
Specimen information			
**Technology**	**ToLID**	**BioSample accession**	**Organism part**
**PacBio long read** **sequencing**	htOpiVive1	SAMEA110137936	Whole organism
**Hi-C sequencing**	htOpiVive2	SAMEA110137937	Whole organism
Sequencing information			
**Platform**	**Run accession**	**Read count**	**Base count (Gb)**
**Hi-C Illumina NovaSeq** **6000**	ERR13361802	8.43e+08	127.26
**PacBio Sequel IIe**	ERR13363382	2.90e+06	22.18

Assembly errors, including 54 missing joins or mis-joins and 6 haplotypic duplications, were corrected by manual curation. This reduced the scaffold number by 2.56% and increased the scaffold N50 by 17.89%. The final assembly has a total length of 627.20 Mb in 342 sequence scaffolds, with 917 gaps, and a scaffold N50 of 175.3 Mb ( Table 2).

**Table 2.** Genome assembly data for *Opisthorchis viverrini*, htOpiVive1.1.

Genome assembly		
Assembly name	htOpiVive1.1	
Assembly accession	GCA_964213165.1	
*Accession of alternate haplotype*	*GCA_964213135.1*	
Span (Mb)	627.20	
Number of contigs	1,260	
Number of scaffolds	342	
Longest scaffold (Mb)	233.41	
Assembly metrics *		*Benchmark*
Contig N50 length (Mb)	1.0	*≥ 1 Mb*
Scaffold N50 length (Mb)	175.3	*= chromosome N50*
Consensus quality (QV)	56.9	*≥ 40*
*k*-mer completeness	Primary: 79.66%, alternate: 77.44%, combined: 98.55%	*≥ 95%*
BUSCO v5.4.3 lineage: metazoa_odb10	C:66.8%[S:66.0%,D:0.8%], F:7.8%,M:25.4%,n:954	*S > 90%*, *D < 5%*
Percentage of assembly mapped to chromosomes	97.89%	*≥ 90%*
Organelles	Mitochondrial genome: 18.04 kb	*complete single alleles*

* Assembly metric benchmarks are adapted from Rhie *et al.* (2021) and the Earth BioGenome Project Report on Assembly Standards September 2024. ** BUSCO scores based on the metazoa_odb10 BUSCO set using version 5.4.3. C = complete [S = single copy, D = duplicated], F = fragmented, M = missing, n = number of orthologues in comparison. A full set of BUSCO scores is available at https://blobtoolkit.genomehubs.org/view/Opisthorchis_viverrini/dataset/GCA_964213165.1/busco.

**Figure 2.**
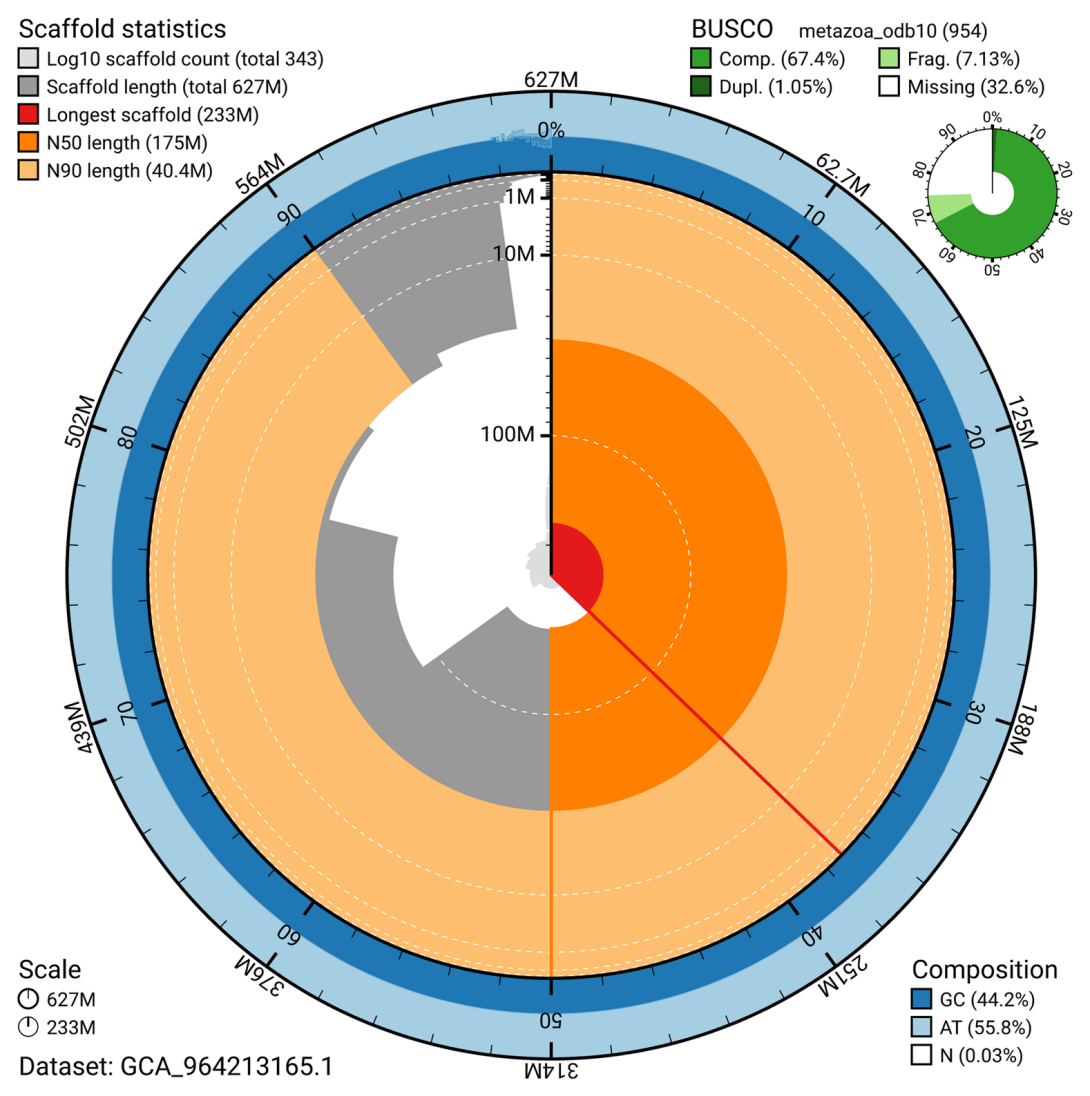
Genome assembly of *Opisthorchis viverrini*, htOpiVive1.1: metrics. The BlobToolKit ‘snail plot’ provides an overview of assembly metrics and BUSCO gene completeness. The circumference represents the length of the whole genome sequence, and the main plot is divided into 1,000 bins around the circumference. The outermost blue tracks display the distribution of GC, AT, and N percentages across the bins. Scaffolds are arranged clockwise from longest to shortest and are depicted in dark grey. The longest scaffold is indicated by the red arc, and the deeper orange and pale orange arcs represent the N50 and N90 lengths. A light grey spiral at the centre shows the cumulative scaffold count on a logarithmic scale. A summary of complete, fragmented, duplicated, and missing BUSCO genes in the metazoa_odb10 set is presented at the top right. An interactive version of this figure is available at https://blobtoolkit.genomehubs.org/view/GCA_964213165.1/dataset/GCA_964213165.1/snail.

**Figure 3.**
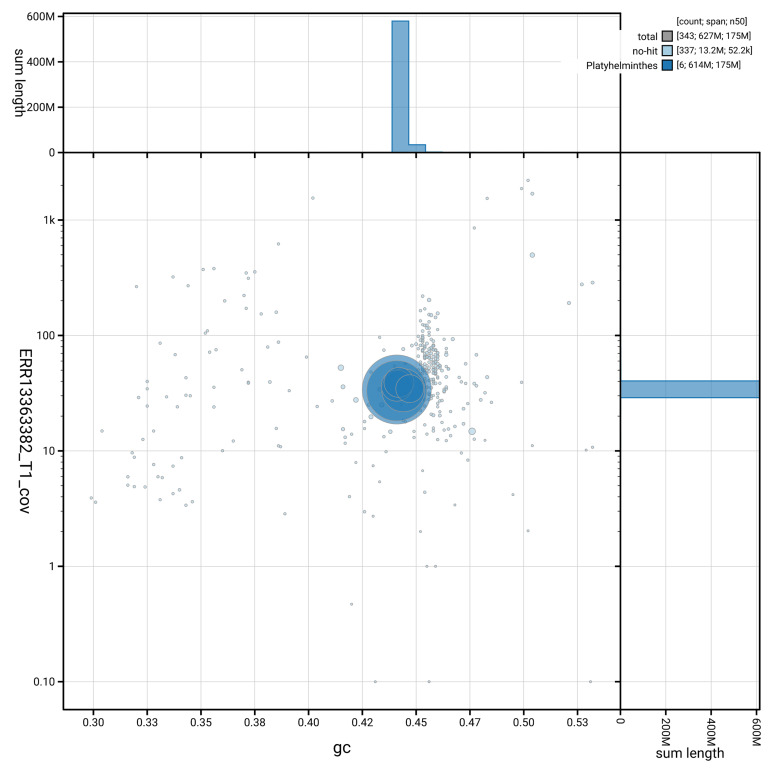
Genome assembly of *Opisthorchis viverrini*, htOpiVive1.1: BlobToolKit GC-coverage plot showing sequence coverage (vertical axis) and GC content (horizontal axis). The circles represent scaffolds, with the size proportional to scaffold length and the colour representing phylum membership. The histograms along the axes display the total length of sequences distributed across different levels of coverage and GC content. An interactive version of this figure is available at https://blobtoolkit.genomehubs.org/view/GCA_964213165.1/dataset/GCA_964213165.1/blob.

**Figure 4.**
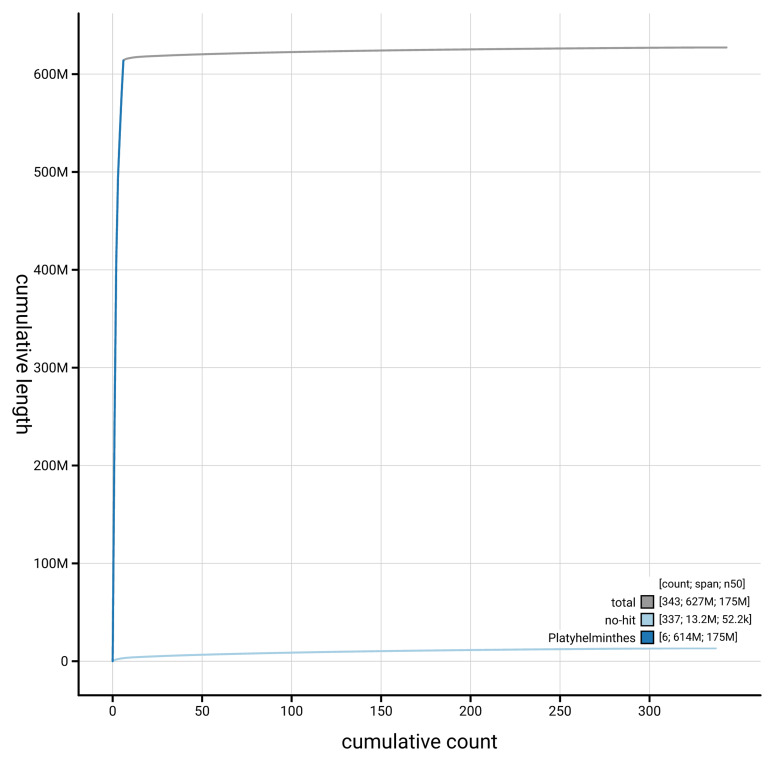
Genome assembly of *Opisthorchis viverrini* htOpiVive1.1: BlobToolKit cumulative sequence plot. The grey line shows cumulative length for all scaffolds. Coloured lines show cumulative lengths of scaffolds assigned to each phylum using the buscogenes taxrule. An interactive version of this figure is available at https://blobtoolkit.genomehubs.org/view/GCA_964213165.1/dataset/GCA_964213165.1/cumulative.

The ‘snail plot’ in Figure 2 provides a summary of the assembly statistics, indicating the distribution of scaffold lengths and other assembly metrics. Figure 3 shows the distribution of scaffolds by GC proportion and coverage. Figure 4 presents a cumulative assembly plot, with separate curves representing different scaffold subsets assigned to various phyla, illustrating the completeness of the assembly.

**Figure 5.**
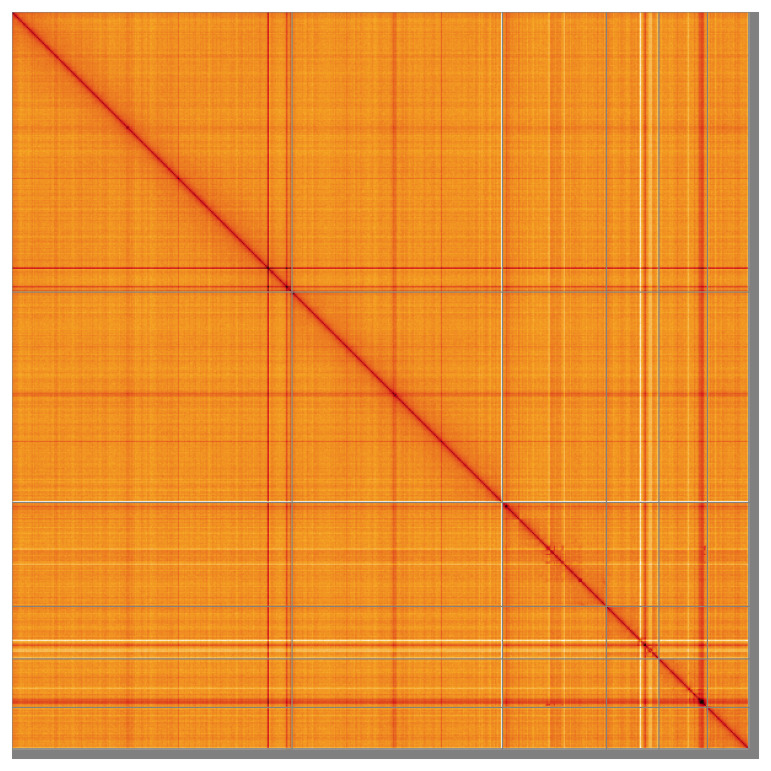
Genome assembly of *Opisthorchis viverrini* htOpiVive1.1: Hi-C contact map of the htOpiVive1.1 assembly, visualised using HiGlass. Chromosomes are shown in order of size from left to right and top to bottom. An interactive version of this figure may be viewed at https://genome-note-higlass.tol.sanger.ac.uk/l/?d=Ehd1atwxSLOgyWAPONN9Nw.

Most of the assembly sequence (97.89%) was assigned to 6 chromosomal-level scaffolds. These chromosome-level scaffolds, confirmed by the Hi-C data, are named in order of size ( Figure 5; Table 3). While not fully phased, the assembly deposited is of one haplotype. Contigs corresponding to the second haplotype have also been deposited. The mitochondrial genome was also assembled and can be found as a contig within the multifasta file of the genome submission, and as a separate fasta file.

**Table 3.** Chromosomal pseudomolecules in the genome assembly of *Opisthorchis viverrini*, htOpiVive1.

INSDC accession	Name	Length (Mb)	GC%
OZ171475.1	1	233.41	44.0
OZ171476.1	2	175.33	44.0
OZ171477.1	3	86.63	44.5
OZ171478.1	4	43.83	44.0
OZ171479.1	5	40.37	44.0
OZ171480.1	6	34.43	44.5
OZ171481.1	MT	0.02	40.5

The final primary assembly has a Quality Value (QV) of 56.9. The *k*-mer completeness for the combined primary and alternate assemblies is 98.55% (79.66% for the primary haplotype and 77.44% for the alternate haplotype). BUSCO (v5.4.3) analysis using the metazoa_odb10 reference set ( *n* = 954) indicated a completeness score of 66.8% (single = 66.0%, duplicated = 0.8%).

## Methods

### Sample acquisition

One specimen of *Opisthorchis viverrini* (specimen ID SAN21000001, ToLID htOpiVive1) was used for genome sequencing, and another (specimen ID SAN21000002, ToLID htOpiVive2) was used for Hi-C sequencing. The specimens (adult worms) were collected from Khon Kaen, Thailand (latitude 16.468, longitude 102.8299) on 2019-06-30 during a curative surgery for a bile duct cancer treatment. The specimens were collected by Narong Kuntikeo and Attapol Titapun (Khon Kaen University) and identified by Paiboon Sithithaworn (Khon Kaen University). The adult worms were frozen at –80 °C immediately following collection and were subsequently shipped to the Wellcome Sanger Institute, Cambridgeshire, UK on 2022-09-28. The parasites were not cultured.

### Nucleic acid extraction

The workflow for high molecular weight (HMW) DNA extraction at the Wellcome Sanger Institute (WSI) Tree of Life Core Laboratory includes a sequence of procedures: sample preparation and homogenisation, DNA extraction, fragmentation and purification. Detailed protocols are available on protocols.io ( Denton *et al.*, 2023b). The htOpiVive1 sample was prepared for DNA extraction by weighing and dissecting it on dry ice ( Jay *et al.*, 2023). Tissue from the whole organism was homogenised using a PowerMasher II tissue disruptor ( Denton *et al.*, 2023a).

HMW DNA was extracted using the Automated MagAttract v2 protocol ( Oatley *et al.*, 2023a). DNA was sheared into an average fragment size of 12–20 kb in a Megaruptor 3 system ( Bates *et al.*, 2023). Sheared DNA was purified by solid-phase reversible immobilisation, using AMPure PB beads to eliminate shorter fragments and concentrate the DNA ( Oatley *et al.*, 2023b). The concentration of the sheared and purified DNA was assessed using a Nanodrop spectrophotometer and Qubit Fluorometer using the Qubit dsDNA High Sensitivity Assay kit. Fragment size distribution was evaluated by running the sample on the FemtoPulse system.

### Hi-C preparation

Tissue from the htOpiVive2 sample was processed at the WSI Scientific Operations core, using the Arima-HiC v2 kit. Tissue (stored at –80 °C) was fixed, and the DNA crosslinked using a TC buffer with 22% formaldehyde. After crosslinking, the tissue was homogenised using the Diagnocine Power Masher-II and BioMasher-II tubes and pestles. Following the kit manufacturer’s instructions, crosslinked DNA was digested using a restriction enzyme master mix. The 5’-overhangs were then filled in and labelled with biotinylated nucleotides and proximally ligated. An overnight incubation was carried out for enzymes to digest remaining proteins and for crosslinks to reverse. A clean up was performed with SPRIselect beads prior to library preparation.

### Library preparation and sequencing

Library preparation and sequencing were performed at the WSI Scientific Operations core. Pacific Biosciences HiFi circular consensus DNA sequencing libraries were prepared using the PacBio Express Template Preparation Kit v2.0 (Pacific Biosciences, California, USA) as per the manufacturer's instructions. The kit includes the reagents required for removal of single-strand overhangs, DNA damage repair, end repair/A-tailing, adapter ligation, and nuclease treatment. Library preparation also included a library purification step using AMPure PB beads (Pacific Biosciences, California, USA) and size selection step to remove templates shorter than 3 kb using AMPure PB modified SPRI. DNA concentration was quantified using the Qubit Fluorometer v2.0 and Qubit HS Assay Kit and the final library fragment size analysis was carried out using the Agilent Femto Pulse Automated Pulsed Field CE Instrument and gDNA 165kb gDNA and 55kb BAC analysis kit. Samples were sequenced using the Sequel IIe system (Pacific Biosciences, California, USA). The concentration of the library loaded onto the Sequel IIe was in the range 40–135 pM. The SMRT link software, a PacBio web-based end-to-end workflow manager, was used to set-up and monitor the run, as well as perform primary and secondary analysis of the data upon completion.

For Hi-C library preparation, DNA was fragmented to a size of 400 to 600 bp using a Covaris E220 sonicator. The DNA was then enriched, barcoded, and amplified using the NEBNext Ultra II DNA Library Prep Kit following manufacturers’ instructions. The Hi-C sequencing was performed using paired-end sequencing with a read length of 150 bp on an Illumina NovaSeq 6000 instrument.

### Genome assembly, curation and evaluation

***Assembly***

The HiFi reads were first assembled using Hifiasm ( Cheng *et al.*, 2021) with the --primary option. Haplotypic duplications were identified and removed using purge_dups ( Guan *et al.*, 2020). The Hi-C reads were mapped to the primary contigs using bwa-mem2 ( Vasimuddin *et al.*, 2019). The contigs were further scaffolded using the provided Hi-C data ( Rao *et al.*, 2014) in YaHS ( Zhou *et al.*, 2023) using the --break option for handling potential misassemblies. The scaffolded assemblies were evaluated using Gfastats ( Formenti *et al.*, 2022), BUSCO ( Manni *et al.*, 2021) and MERQURY.FK ( Rhie *et al.*, 2020).

The mitochondrial genome was assembled using MitoHiFi ( Uliano-Silva *et al.*, 2023), which runs MitoFinder ( Allio *et al.*, 2020) and uses these annotations to select the final mitochondrial contig and to ensure the general quality of the sequence.

***Assembly curation***

The assembly was decontaminated using the Assembly Screen for Cobionts and Contaminants (ASCC) pipeline (article in preparation). Flat files and maps used in curation were generated in TreeVal ( Pointon *et al.*, 2023). Manual curation was primarily conducted using PretextView ( Harry, 2022), with additional insights provided by JBrowse2 ( Diesh *et al.*, 2023) and HiGlass ( Kerpedjiev *et al.*, 2018). Scaffolds were visually inspected and corrected as described by Howe *et al*. (2021). Any identified contamination, missed joins, and mis-joins were corrected, and duplicate sequences were tagged and removed. The curation process is documented at https://gitlab.com/wtsi-grit/rapid-curation (article in preparation).

***Assembly quality assessment***

The Merqury.FK tool ( Rhie *et al.*, 2020) was used to evaluate *k*-mer completeness and assembly quality for the primary and alternate haplotypes using the *k*-mer databases ( *k* = 31) that were pre-computed prior to genome assembly. The analysis outputs included assembly QV scores and completeness statistics.

A Hi-C contact map was produced for the final, public version of the assembly. The Hi-C reads were aligned using bwa-mem2 ( Vasimuddin *et al.*, 2019) and the alignment files were combined using SAMtools ( Danecek *et al.*, 2021). The Hi-C alignments were converted into a contact map using BEDTools ( Quinlan & Hall, 2010) and the Cooler tool suite ( Abdennur & Mirny, 2020). The contact map is visualised in HiGlass ( Kerpedjiev *et al.*, 2018).

The blobtoolkit pipeline is a Nextflow port of the previous Snakemake Blobtoolkit pipeline ( Challis *et al.*, 2020). It aligns the PacBio reads in SAMtools and minimap2 ( Li, 2018) and generates coverage tracks for regions of fixed size. In parallel, it queries the GoaT database ( Challis *et al.*, 2023) to identify all matching BUSCO lineages to run BUSCO ( Manni *et al.*, 2021). For the three domain-level BUSCO lineages, the pipeline aligns the BUSCO genes to the UniProt Reference Proteomes database ( Bateman *et al.*, 2023) with DIAMOND blastp ( Buchfink *et al.*, 2021). The genome is also divided into chunks according to the density of the BUSCO genes from the closest taxonomic lineage, and each chunk is aligned to the UniProt Reference Proteomes database using DIAMOND blastx. Genome sequences without a hit are chunked using seqtk and aligned to the NT database with blastn ( Altschul *et al.*, 1990). The blobtools suite combines all these outputs into a blobdir for visualisation.

The genome assembly and evaluation pipelines were developed using nf-core tooling ( Ewels *et al.*, 2020) and MultiQC ( Ewels *et al.*, 2016), relying on the Conda package manager, the Bioconda initiative ( Grüning *et al.*, 2018), the Biocontainers infrastructure ( da Veiga Leprevost *et al.*, 2017), as well as the Docker ( Merkel, 2014) and Singularity ( Kurtzer *et al.*, 2017) containerisation solutions.

Table 4 contains a list of relevant software tool versions and sources.

**Table 4.** Software tools: versions and sources.

Software tool	Version	Source
BEDTools	2.30.0	https://github.com/arq5x/bedtools2
BLAST	2.14.0	http://ftp.ncbi.nlm.nih.gov/blast/executables/blast+/
BlobToolKit	4.3.7	https://github.com/blobtoolkit/blobtoolkit
BUSCO	5.4.3 and 5.5.0	https://gitlab.com/ezlab/busco
bwa-mem2	2.2.1	https://github.com/bwa-mem2/bwa-mem2
Cooler	0.8.11	https://github.com/open2c/cooler
DIAMOND	2.1.8	https://github.com/bbuchfink/diamond
fasta_ windows	0.2.4	https://github.com/tolkit/fasta_windows
FastK	427104ea91c78c3b8b8b49f1a7d6bbeaa869ba1c	https://github.com/thegenemyers/FASTK
Gfastats	1.3.6	https://github.com/vgl-hub/gfastats
GoaT CLI	0.2.5	https://github.com/genomehubs/goat-cli
Hifiasm	0.19.8-r587	https://github.com/chhylp123/hifiasm
HiGlass	44086069ee7d4d3f6f3f0012569789ec138f42b84aa 44357826c0b6753eb28de	https://github.com/higlass/higlass
Merqury.FK	d00d98157618f4e8d1a9190026b19b471055b22e	https://github.com/thegenemyers/MERQURY.FK
MitoHiFi	3	https://github.com/marcelauliano/MitoHiFi
MultiQC	1.14, 1.17, and 1.18	https://github.com/MultiQC/MultiQC
NCBI Datasets	15.12.0	https://github.com/ncbi/datasets
Nextflow	23.04.0-5857	https://github.com/nextflow-io/nextflow
PretextView	0.2.5	https://github.com/sanger-tol/PretextView
purge_dups	1.2.5	https://github.com/dfguan/purge_dups
samtools	1.16.1, 1.17, and 1.18	https://github.com/samtools/samtools
sanger-tol/ ascc	-	https://github.com/sanger-tol/ascc
Seqtk	1.3	https://github.com/lh3/seqtk
Singularity	3.9.0	https://github.com/sylabs/singularity
TreeVal	1.0.0	https://github.com/sanger-tol/treeval
YaHS	1.2a.2	https://github.com/c-zhou/yahs

### Wellcome Sanger Institute – Legal and Governance

The materials that have contributed to this genome note have been supplied by a Tree of Life collaborator. The Wellcome Sanger Institute employs a process whereby due diligence is carried out proportionate to the nature of the materials themselves, and the circumstances under which they have been/are to be collected and provided for use. The purpose of this is to address and mitigate any potential legal and/or ethical implications of receipt and use of the materials as part of the research project, and to ensure that in doing so we align with best practice wherever possible.

The overarching areas of consideration are:

•   Ethical review of provenance and sourcing of the material

•   Legality of collection, transfer and use (national and international)

Each transfer of samples is undertaken according to a Research Collaboration Agreement or Material Transfer Agreement entered into by the Tree of Life collaborator, Genome Research Limited (operating as the Wellcome Sanger Institute) and in some circumstances other Tree of Life collaborators.

## Data Availability

European Nucleotide Archive: *Opisthorchis viverrini* (Asian liver fluke). Accession number PRJEB67413; https://identifiers.org/ena.embl/PRJEB67413. The genome sequence is released openly for reuse by the Wellcome Sanger Institute Tree of Life Programme ( https://www.sanger.ac.uk/programme/tree-of-life/). All raw sequence data and the assembly have been deposited in INSDC databases. The genome will be annotated using available RNA-Seq data and presented through the Ensembl pipeline at the European Bioinformatics Institute. Raw data and assembly accession identifiers are reported in Table 1 and Table 2.

## Author information

Members of the Wellcome Sanger Institute Tree of Life Management, Samples and Laboratory team are listed here: https://doi.org/10.5281/zenodo.12162482.

Members of Wellcome Sanger Institute Scientific Operations: Sequencing Operations are listed here: https://doi.org/10.5281/zenodo.12165051.

Members of the Wellcome Sanger Institute Tree of Life Core Informatics team are listed here: https://doi.org/10.5281/zenodo.12160324.

Members of the Tree of Life Core Informatics collective are listed here: https://doi.org/10.5281/zenodo.12205391.
