# Supplementary material for: The genome sequence of the liver fluke Opisthorchis viverrini (Poirier, 1886) Stiles & Hassall, 1896
Source: Wellcome Open Res. 2025 Jan 10;10:1. [Version 1] doi: 10.12688/wellcomeopenres.23535.1 (PMC11822251; doi:10.12688/wellcomeopenres.23535.1)
Supplement: Peer review history

# Reviewer report: Reviewer response for version 1

**DOI:** 10.21956/wellcomeopenres.25958.r117152

**Published:** 2025-02-12

**Authors:** Ferdinand Marlétaz

**Affiliations:** 1 Centre for Life’s Origin & Evolution, Department of Ecology, Evolution & Environment, University College London, London, UK

The manuscript reports the genome of the liver fluke *Opisthorchis viverrini *sequenced at the Sanger institute assembled in 6 putative chromosomes  (233Mb). Relevant and interesting background is provided. The assembly was performed using standard and established procedures and appears sufficiently described. As a comment that I made repeatedly for similar reports, the contact maps appears difficult to read regarding chromosomal identity and contacted and some improvement in the plotting would be desirable.

Are sufficient details of methods and materials provided to allow replication by others?

Yes

Is the rationale for creating the dataset(s) clearly described?

Yes

Are the datasets clearly presented in a useable and accessible format?

Yes

Are the protocols appropriate and is the work technically sound?

Yes

Reviewer Expertise:

comparative genomics, phylogeny of animals, evodevo

I confirm that I have read this submission and believe that I have an appropriate level of expertise to confirm that it is of an acceptable scientific standard.

# Reviewer report: Reviewer response for version 1

**DOI:** 10.21956/wellcomeopenres.25958.r117156

**Published:** 2025-02-10

**Authors:** Jose Tort

**Affiliations:** 1 Genetics, Universidad de la República, Montevideo, Montevideo, 11800, Uruguay

The article describes the sequencing and assembly at chromosome level of the genome of the parasitic trematode *Opisthorchis viverrini*.  The procedures are appropriate for the study, the quality of assembly obtained is very good according to provided quality parameters. The procedures and general characteristics of the assembly generated are clear.

 There is no annotation associated with this assembly and is reported that this will be done using available RNA-seq data. 

 The data has been deposited (and are available) at the ENA. Is also reported that the sequences are released at the  Tree of Life program but the link  provided lead to general information of the program and not to the data. In fact after crawling within the site I could not find the data. 

 Rather than these, the natural place to locate the data is WormBase Parasite, a well established and widely used database of genomes from diverse helminths, This is the natural place for the research community to seek information on parasitic helminths genomes and associated data (transcriptomes, proteomes, etc). 

 Contributing to it would help to maintain a valuable resource much appreciated by the community.

Are sufficient details of methods and materials provided to allow replication by others?

Yes

Is the rationale for creating the dataset(s) clearly described?

Yes

Are the datasets clearly presented in a useable and accessible format?

Partly

Are the protocols appropriate and is the work technically sound?

Yes

Reviewer Expertise:

Parasite molecular biologiy and genomics

I confirm that I have read this submission and believe that I have an appropriate level of expertise to confirm that it is of an acceptable scientific standard.
